# Taxonomic notes on the Chinese species of the genus *Broscosoma* Rosenhauer, 1846 (Coleoptera, Carabidae, Broscinae), with description of seven new species

**DOI:** 10.3897/zookeys.1285.190753

**Published:** 2026-07-21

**Authors:** Ri-Xin Jiang, Jia-Heng Chen, Zi-Zhong Li, Xiang-Sheng Chen

**Affiliations:** 1 Guizhou Key Laboratory of Agricultural Biosecurity, Guizhou University, Guiyang, 550025, Guizhou, China Guizhou Key Laboratory of Agricultural Biosecurity, Guizhou University Guiyang China; 2 Institute of Entomology, Guizhou University, Guiyang, 550025, Guizhou, China Institute of Entomology, Guizhou University Guiyang China; 3 The Provincial Special Key Laboratory for Development and Utilization of Insect Resources, Guizhou University, Guiyang, 550025, Guizhou, China College of Forestry, Beijing Forestry University Beijing China; 4 College of Forestry, Beijing Forestry University, Beijing, 100083, China The Provincial Special Key Laboratory for Development and Utilization of Insect Resources, Guizhou University Guiyang China

**Keywords:** Broscini, China, diagnostic features, ground beetle, morphology

## Abstract

The ground beetle genus *Broscosoma* Rosenhauer, 1846 comprises 62 described species, distributed in Western Europe, East Asia, and its adjacent regions. China exhibits particularly high species diversity within this genus, with 45 recorded species and subspecies. In this study, we describe seven new *Broscosoma* species from China: *B. chaoshan***sp. nov**. from Guangdong Province, *B. epanggong***sp. nov**. from Sichuan Province, *B. jiangyuxini***sp. nov**. from Guizhou Province, *B. luojingxiangi***sp. nov**. from Zhejiang Province, *B. surongxiangi***sp. nov**. from Fujian Province, *B. xumaozhoui***sp. nov**. from Hubei Province and Chongqing City, and *B. zhouhanpingi***sp. nov**. from Chongqing City. Habitus and diagnostic features of the new species are illustrated, along with a checklist of all known Chinese *Broscosoma* species and a distribution map of Chinese *Broscosoma* species outside the Qinghai-Xizang Plateau and Hengduan Mountains region. The comparative diagnoses discuss characters of the new and known species. The results indicate that the existing species diversity requires more detailed research focusing on larger areas of South China in the future.

## Introduction

The Broscini genus *Broscosoma* Rosenhauer, 1846 currently includes 62 valid species. The distribution of the genus is widely disjunctive, with two species found in the Italian Alps, one species in the Caucasus, and all the remaining species distributed in East Asia and its adjacent regions (northern India, Nepal), especially showing relatively high species diversity in the Himalayan and Hengduan Mountains regions (Bousquet 2003; Sciaky and Facchini 2005; Barševskis 2010; Häckel et al. 2010; Deuve 2011a, 2011b, 2014, 2018, 2023; Häckel 2017; Jiang et al. 2020, 2021; Kavanaugh and Liang 2021; Jiang and Chen 2023; Deuve et al. 2025).

China harbors a notably high diversity of *Broscosoma*, particularly in the Hengduan Mountains region (Yunnan and western Sichuan Provinces), with 45 species recorded in the country to date (Bousquet 2003; Sciaky and Facchini 2005; Häckel et al. 2010; Häckel 2017; Jiang et al. 2020, 2021; Kavanaugh and Liang 2021; Deuve 2023; Jiang and Chen 2023; Deuve et al. 2025). Historically, the genus *Broscosoma* was long considered to be restricted to the high-altitude areas of the Palearctic (Sciaky and Facchini 2005; Häckel et al. 2010; Häckel 2017; Deuve et al. 2025). However, recent research has revealed that species of this genus can also be found in lower-altitude forest environments of the subtropics (Jiang et al. 2020, 2021; Jiang and Chen 2023).

Many Chinese *Broscosoma* species are highly localized, and vast areas of southern China still lack systematic surveys. As a result, the true diversity of the genus *Broscosoma* in China remains poorly understood (Deuve et al. 2025). For example, a large number of *Broscosoma* species were discovered in a particular area of the Gaoligong Mountains during intensive research, most of which are new to science (Kavanaugh and Liang 2021).

In the present article, we describe seven new *Broscosoma* species from China: *B. chaoshan* sp. nov. from Guangdong Province, *B. epanggong* sp. nov. from Sichuan Province, *B. jiangyuxini* sp. nov. from Guizhou Province, *B. luojingxiangi* sp. nov. from Zhejiang Province, *B. surongxiangi* sp. nov. from Fujian Province, *B. xumaozhoui* sp. nov. from Hubei Province and Chongqing City, and *B. zhouhanpingi* sp. nov. from Chongqing City. The habitus and diagnostic features of the new species are illustrated. In addition, a checklist of all known Chinese *Broscosoma* species (Table 1) and a distribution map of the species outside the Qinghai-Xizang Plateau and Hengduan Mountains region are provided (Fig. 19).

**Table 1.** List of Chinese *Broscosoma* species.

**Species (Chinese common name)**	**Distribution**	**Reference**
*B. bicoloratum* Kavanaugh & Liang, 2021 (双色珠步甲)	Yunnan	Kavanaugh and Liang (2021)
*B. bolaicum* Deuve, Liang & Shi, 2025 (博拉珠步甲)	Xizang	Deuve et al. (2025)
*B. businskae* Dvořák, 1998 (布氏珠步甲)	Xizang	Dvořák (1998), Sciaky and Facchini (2005)
*B. chaoshan* sp. nov. (潮汕珠步甲)	Guangdong	This paper
*B. danbaense* Deuve, 2014 (丹巴珠步甲)	Sichuan	Deuve (2014)
*B. danzhuensis* Kavanaugh & Liang, 2021 (丹珠珠步甲)	Yunnan	Kavanaugh and Liang (2021)
*B. dostali* Deuve, 2006 (多氏珠步甲)	Sichuan	Deuve (2006b)
*B. dostalianum* Deuve, 2014 (铜绿珠步甲)	Sichuan	Deuve (2014)
*B. epanggong* sp. nov. (阿房宫珠步甲)	Sichuan	This paper
*B. farkaci* Sciaky & Facchini, 2005 (法氏珠步甲)	Xizang	Sciaky and Facchini (2005), Deuve et al. (2025)
*B. furvum* Kavanaugh & Liang, 2021 (暗珠步甲)	Yunnan	Kavanaugh and Liang (2021)
*B. gaoligongense* Deuve & Wrase, 2015 (高黎贡珠步甲)	Yunnan	Deuve and Wrase (2015)
*B. gongshanense* Kavanaugh & Liang, 2021 (贡山珠步甲)	Yunnan	Kavanaugh and Liang (2021)
*Broscosoma gracile* Andrewes, 1927 (纤珠步甲)	China: Xizang; India	Andrewes (1927); Deuve et al. (2025)
*B. guoliangi* Jiang, Liu & Wang, 2021 (郭亮珠步甲)	Fujian	Jiang et al. (2021)
*B. herculeanum* Deuve, 2011 (力神珠步甲)	Sichuan	Deuve (2011a)
*B. holomarginatum* Kavanaugh & Liang, 2021 (全缘珠步甲)	Yunnan, Xizang	Kavanaugh and Liang (2021), Deuve et al. (2025)
*B. janatai* Deuve, 2008 (亚氏珠步甲)	Sichuan	Deuve (2008b)
*B. jiangyuxini* sp. nov. (姜氏珠步甲)	Guizhou	This paper
*B. jintangense* Deuve, 2008 (金汤珠步甲)	Sichuan	Deuve (2008a)
*B. kalabi kalabi* Deuve, 1992 (卡氏珠步甲指名亚种)	Sichuan	Deuve (1992), Sciaky and Facchini (2005)
*B. kalabi xiaojinense* Deuve, 1994 (卡氏珠步甲小金亚种)	Sichuan	Deuve (2014), Deuve (2023)
*B. kalabianum kalabianum* Deuve, 2014 (蜀珠步甲指名亚种)	Sichuan	Deuve (2014)
*B. kalabianum viridescens* Deuve, 2018 (蜀珠步甲暗绿亚种)	Sichuan	Deuve (2018)
*B. luojingxiangi* sp. nov. (罗氏珠步甲)	Zhejiang	This paper
*B. mirabile* Deuve, Liang & Shi, 2025 (美珠步甲)	Sichuan	Deuve et al. (2025)
*B. montreuili* Deuve, 2006 (蒙氏珠步甲)	Sichuan	Deuve (2006b)
*B. moriturum* Semenov, 1900 (珍珠步甲)	Sichuan	Semenov (1900), Sciaky and Facchini (2005)
*B. mourzinei* Deuve, 2011 (穆氏珠步甲)	Sichuan	Deuve (2011b)
*B. parvulum* Deuve, 2023 (微珠步甲)	Gansu	Deuve (2023)
*B. parvum* Kavanaugh & Liang, 2021 (小珠步甲)	Yunnan	Kavanaugh and Liang (2021)
*B. purpureum* Kavanaugh & Liang, 2021 (蓝紫珠步甲)	Yunnan	Kavanaugh and Liang (2021)
*B. qiului* Jiang, Liu & Wang, 2021 (邱鹭珠步甲)	Chongqing	Jiang et al. (2021)
*B. resbecqi* Kavanaugh & Liang, 2021 (雷氏珠步甲)	Yunnan	Kavanaugh and Liang (2021)
*B. ribbei* Putzeys, 1877 (翡翠珠步甲)	China: Yunnan, Xizang; India, Nepal, Pakistan	Putzeys (1877), Deuve and Tian (2002), Sciaky and Facchini (2005), Jiang et al. (2021), Kavanaugh and Liang (2021), Deuve et al. (2025)
*B. sehnali* Deuve, 2006 (塞氏珠步甲)	Sichuan	Deuve (2006a)
*B. shizishanense* Deuve, Liang & Shi, 2025 (狮子山珠步甲)	Sichuan	Deuve et al. (2025)
*B. sichuanum* Deuve, 1990 (四川珠步甲)	Sichuan	Deuve (1990), Sciaky and Facchini (2005)
*B. stefani stefani* Sciaky & Facchini, 2005 (斯氏珠步甲指名亚种)	Sichuan	Sciaky and Facchini (2005)
*B. stefani fubianense* Deuve, 2014 (斯氏珠步甲抚边亚种)	Sichuan	Deuve (2014)
*B. surongxiangi* sp. nov. (苏氏珠步甲)	Fujian	This paper
*B. tiani* Deuve, 2006 (田氏珠步甲)	Sichuan	Deuve (2006b)
*B. tibetanum* Facchini, 2002 (藏珠步甲)	Xizang	Facchini (2002), Sciaky and Facchini (2005)
*B. uenoi* Habu, 1972 (上野珠步甲)	Taiwan	Habu (1973), Sciaky and Facchini (2005)
*B. valainisi* Barševskis, 2010 (瓦氏珠步甲)	Shaanxi	Barševskis (2010), Li and Chen (2022)
*B. viridicollare* Kavanaugh & Liang, 2021 (绿缘珠步甲)	Yunnan	Kavanaugh and Liang (2021)
*B. wuyishanum* Jiang & Chen, 2023 (武夷山珠步甲)	Fujian	Jiang and Chen (2023)
*B. xuechengense* Deuve, 2008 (薛城珠步甲)	Sichuan	Deuve (2008a)
*B. xuhaoi* Jiang, Feng & Wang, 2020 (许浩珠步甲)	Chongqing	Jiang et al. (2020)
*B. xumaozhoui* sp. nov. (徐氏珠步甲)	Hubei, Chongqing	This paper
*B. zhengyuandongi* Jiang, Feng & Wang, 2020 (郑氏珠步甲)	Fujian	Jiang et al. (2020)
*B. zhouhanpingi* sp. nov. (周氏珠步甲)	Chongqing	This paper

## Materials and methods

The material examined during this work is deposited in the Institute of Entomology, Guizhou University, Guiyang, China (**GUGC**) and the Forest Entomology Laboratory, Beijing Forestry University, Beijing, China (**CBFU**).

Collecting data of the specimens are quoted verbatim. The Chinese translation of each locality below the provincial level is included in parentheses at the first appearance in the text. Each type specimen bears the following label: ‘HOLOTYPE (red) (or PARATYPE (yellow)), ♂ (or ♀), *Broscosoma* + specific name sp. nov., Jiang, Chen, Li & Chen, 2026.’.

Images of the habitus and the morphological details were taken using a Canon 5D Mark IV digital camera with an MP-E 65 mm f/2.8 1–5× macro lens. A Godox MF12 flash was used as the light source. Images of the aedeagus, parameres and ovipositor were taken using a Nikon SMZ25 stereoscopic microscope with a Nikon DS-Ri2 camera. Zerene Stacker (v. 1.04) was used for image stacking. All images were improved and grouped into plates in Adobe Photoshop CS5 Extended.

The following abbreviations are applied: **HL**—length of head from the anterior clypeal margin to the occipital constriction; **HW**—width of head across eyes; PL—length of pronotum along the midline; **PW**—maximum width of pronotum; **EL**—length of elytra from base of scutellum to apex of suture; **EW**—maximum width of elytra; **BL**—length of body (sum of HL + PL + EL).

## Taxonomic account

### Broscosoma chaoshan sp. nov.

Taxon classificationAnimaliaColeopteraCarabidae

D0E5FA4F-3C03-5298-82CE-D5AFE336E086

https://zoobank.org/39CEB8D1-6B1A-4CFF-986C-B353230F3217

Figs 1, 2, 16 C

#### Common name.

潮汕珠步甲.

#### Type material

(1 ♂, 2 ♀♀): ***Holotype***: China: • ♂, labeled ‘China: Guangdong Province (广东省), Chaozhou City (潮州市), Chaoan District (潮安区), Fenghuang Town (凤凰镇), Fenghuangshan Mts (凤凰山), H: 1300 m, 02.VI.2024’ (GUGC). ***Paratypes***: • 2 ♀♀, labeled ‘China: Guangdong Province (广东省), Chaozhou City (潮州市), Chaoan District (潮安区), Fenghuangshan Mts (凤凰山), H: 1000 m, 11.X.2020, Huan-Tong Chen & Xu-Tong Lin leg.’ (CBFU).

#### Diagnosis.

Size small to medium-sized, body pyriform; dorsal surface shiny, without metallic luster, dark brown to black; legs, labrum, palpi and antennae reddish brown. Pronotum with basal portion smooth. Elytra with eight distinct striae, all of them strongly incised and coarsely punctate; shoulders markedly rounded. Aedeagus with apical 1/3 strongly narrowed, with apex nearly truncated; apical part of left paramere nearly straight, tip with several short setae.

#### Description.

**Figure 1.**
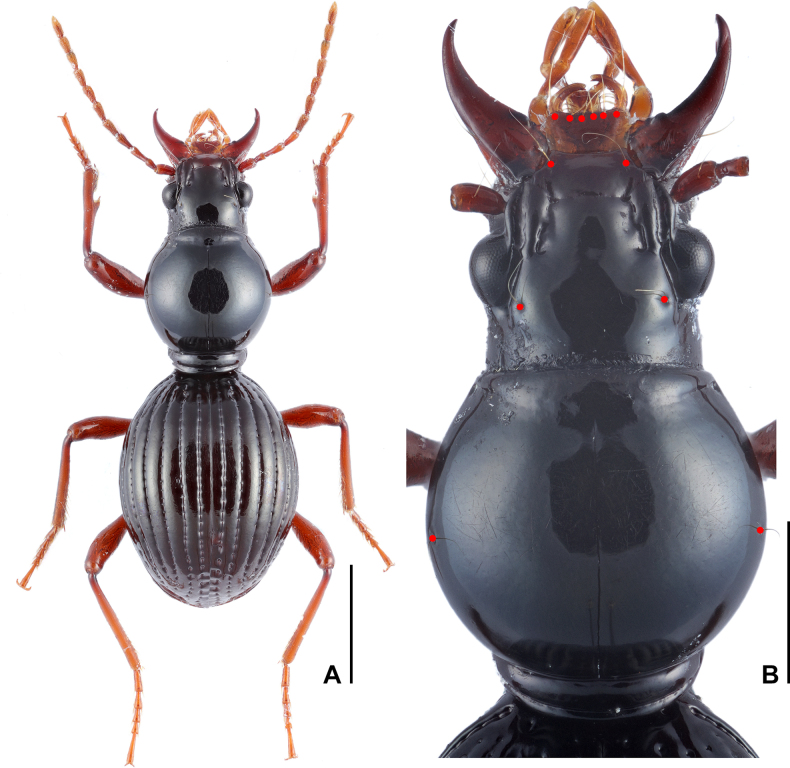
**A**. Dorsal habitus of *Broscosoma chaoshan* sp. nov., male; **B**. Ditto, head and pronotum, dorsal view. Scale bars: 2 mm (**A**); 1 mm (**B**). Red dots: setigerous punctures.

**Male. *Body*** (Fig. 1A) pyriform; dorsal surface dark brown to black, much shiny, without metallic luster; legs, labrum, palpi and antennae reddish brown.

***Head*** (Fig. 1B) finely convex; dorsal surface shiny and smooth, without micro-punctures; labrum nearly rectangular, with six setae and weakly concave at apical margin; frontal grooves irregular and impunctate; apical margin of clypeus slightly convex with one seta on each side; eyes large, nearly hemispherical; tempora very shortly and weakly swollen.

***Pronotum*** (Fig. 1B) globose, distinctly convex, approximately as long as wide, with maximum width slightly anterior to the middle, equally constricted at anterior and posterior margins; dorsal surface without micro-punctures; median longitudinal impression and anterior transverse impression shallow but distinct; one mid-lateral seta present at anterior middle of pronotum on each side, basal-lateral seta absent; basal portion smooth, without punctures or rugoses.

**Figure 2.**
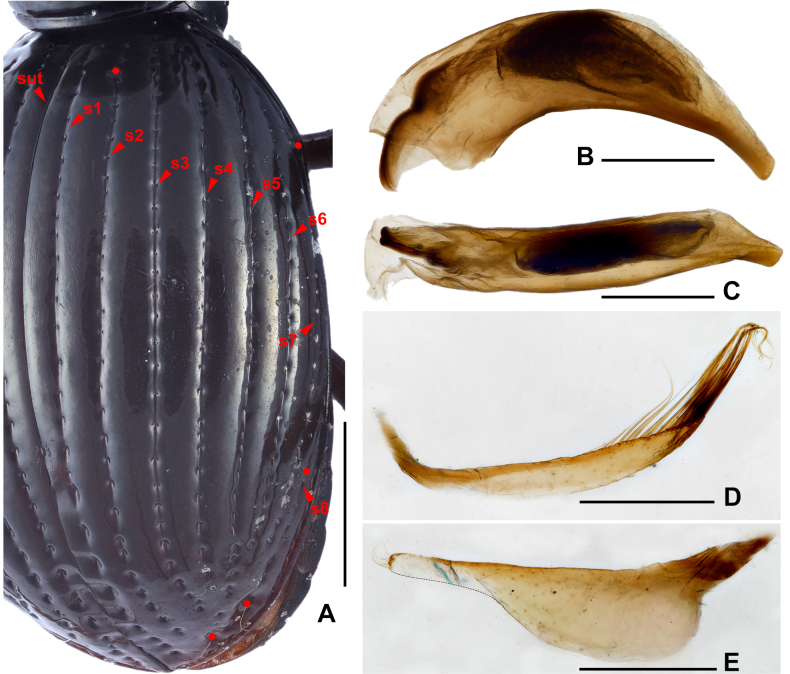
Diagnostic features of *Broscosoma chaoshan* sp. nov., male. **A**. Elytra, oblique right lateral view; **B**. Median lobe of aedeagus, lateral view; **C**. Ditto, ventral view; **D**. Right paramere; **E**. Left paramere. Scale bars: 1 mm (**A**); 0.5 mm (**B–E**). Features in red: sut, elytral suture; s1–s8, elytral striae 1–8. Red dots: setigerous punctures.

***Elytra*** (Fig. 2A) ovoid, strongly convex; shoulders extremely narrow and rounded; each elytron with eight distinct striae, all striae deeply incised and coarsely punctate; one parascutellar seta present at base of stria 2, discal setae absent, umbilicate series comprised of one humeral, one preapical and two apical pores.

***Legs*** simple, glabrous, only tibial apex with a few thin setae, protarsomeres 1–4 with adhesive setae ventrally.

***Median lobe of aedeagus*** (Fig. 2B, C) simple, apical 1/3 strongly narrowed, ended in a nearly truncate tip in lateral view, slender and weakly curved in ventral view. Left paramere (Fig. 2E) expanded at middle, apical part nearly straight, with several short setae at tip; right paramere (Fig. 2D) slender, curved, with dense long setae along apico-anterior margin.

**Female**: externally similar to the male, protarsomeres 1–4 without adhesive setae ventrally.

#### Measurements

**(mm)**. Males: BL 7.41; HL 1.17, HW 1.56; PL 2.16, PW 2.18; EL 4.08, EW 3.05. Females: BL 7.87–8.02; HL 1.28–1.40, HW 1.53–1.61; PL 2.49–2.59, PW 2.22–2.28; EL 4.00–4.13, EW 3.09–3.10.

#### Comparison.

Members of the new species are most similar to those of *B. zhengyuandongi*, *B. jiangyuxini* sp. nov. and *B. zhouhanpingi* sp. nov. Members of these four species differ from those of other species in the combination of following characters: 1) small-sized and pyriform body; 2) dorsal surface without metallic luster; 3) each elytron with eight distinct striae, all striae distinctly incised. Among these species, members of *B. chaoshan* sp. nov. are most similar to those of *B. zhengyuandongi*, and distinctly differ from those of the latter two species in having: 1) pronotal base smooth (vs faintly wrinkled in *B. jiangyuxini* sp. nov., sparsely and finely punctate in *B. zhouhanpingi* sp. nov.); 2) elytral striae quite deeply incised and coarsely punctate (vs striae less incised and finely punctate in the latter two); 3) the different form of aedeagus.

*Broscosoma chaoshan* sp. nov. members can be readily distinguished from *B. zhengyuandongi* members by the following characters: 1) eyes larger, nearly hemispherical (vs eyes medium-sized in *B. zhengyuandongi*); 2) tempora very shortly and faintly swollen (vs tempora short but distinctly swollen behind eyes); 3) median lobe of aedeagus with apical 1/3 strongly narrowed, ended in a nearly truncate tip (vs ended in a widely rounded tip in *B. zhengyuandongi*); 4) apical part of left paramere nearly straight, with several short setae at tip (vs apical part of left paramere distinctly curved, without setae at tip).

#### Distribution.

China: only known from the type locality, Mts. Fenghuangshan in the eastern part of Guangdong Province.

#### Biological notes.

All specimens were collected on the relatively low area of a damp cliff at night (Fig. 16C).

#### Etymology.

The specific name *chaoshan* is a cultural and geographical region in the eastern part of Guangdong Province, China, typically referring to the three cities of Shantou, Chaozhou (the type locality of this new species), and Jieyang. The name is treated as a noun in apposition.

### Broscosoma epanggong sp. nov.

Taxon classificationAnimaliaColeopteraCarabidae

0FF54302-4551-5624-8F64-43BB9D0C9E03

https://zoobank.org/93443AE0-3ACB-4126-BC06-08B7B29E9742

Figs 3, 4, 16 A, B

#### Common name.

阿房宫珠步甲.

#### Type material

(3 ♂♂): ***Holotype***: China: • ♂, labeled ‘China: Sichuan Province (四川省), Bazhong City (巴中市), Nanjiang County (南江县), Mts. Guangwushan (光雾山), Yanziling (燕子岭), 32.690778°N, 106.800319°E, H: 1706 m, 27.VI.2024, Mao-Zhou Xu, Yu-Zhou Huang & Yi-Teng Li leg.’ (CBFU). ***Paratypes***: • 2 ♂♂, with the same label data as the holotype (CBFU).

#### Diagnosis.

Size small, body elongate-pyriform; dorsal surface shiny with weak metallic luster, dark brown to black; labrum, palpi and antennae reddish brown; legs yellowish brown. Elytral stria 1 relatively incised, striae 2–6 distinct but shallow, striae 7 and 8 very short and shallow, all striae finely punctate; shoulders distinctly rounded. Basal portion of pronotum sparsely and coarsely punctate, with just a few punctures connected forming short wrinkles. Aedeagus strongly curved and ended in a rounded tip; left paramere with very short setae at apex of anterior margin.

#### Description.

**Figure 3.**
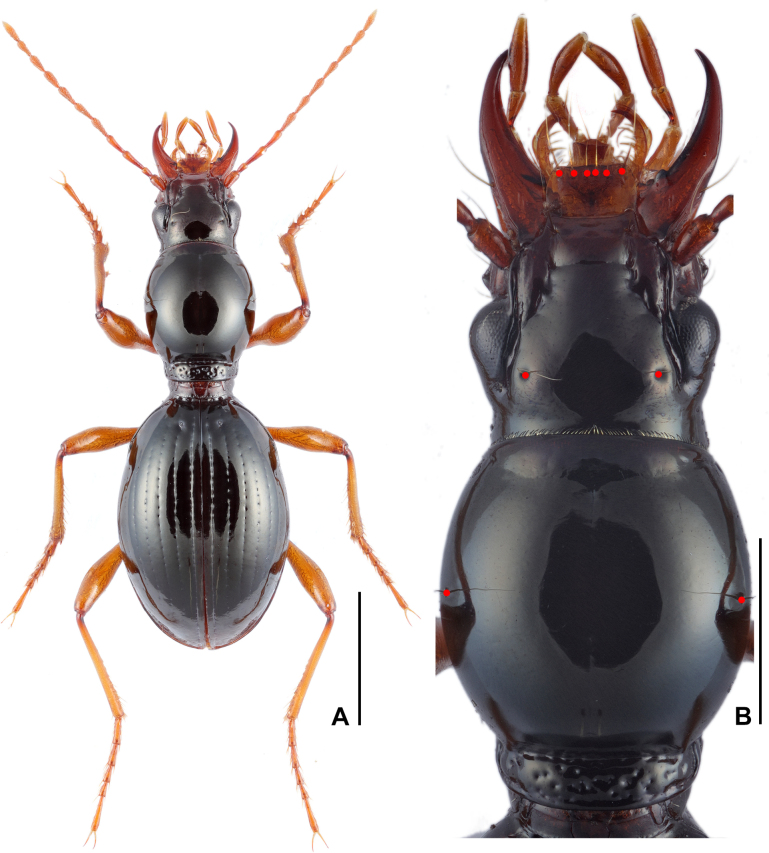
**A**. Dorsal habitus of *Broscosoma epanggong* sp. nov., male; **B**. Ditto, head and pronotum, dorsal view. Scale bars: 2 mm (**A**); 1 mm (**B**). Red dots: setigerous punctures.

**Male. *Body*** (Fig. 3A) elongate-pyriform; dorsal surface dark brown to black, shiny with faint metallic luster; labrum, palpi and antennae reddish brown; legs yellowish brown.

***Head*** (Fig. 3B) finely convex, dorsal surface shiny, dorsal surface shiny and smooth; frontal grooves irregular and impunctate; labrum nearly rectangular, with six setae and faintly curved at apical margin; frontal grooves irregular and impunctate; apical margin of clypeus curved with one seta on each side; eyes large, moderately convex.

***Pronotum*** (Fig. 3B) globose, distinctly convex, longer than wide, with maximum width anterior to the middle, equally constricted at anterior and posterior margins; dorsal surface without micro-punctures; median longitudinal impression and anterior transverse impression shallow but distinct; one mid-lateral seta present at anterior middle of pronotum on each side, basal-lateral seta absent; basal portion sparsely and coarsely punctate, with few punctures connected forming short wrinkle.

**Figure 4.**
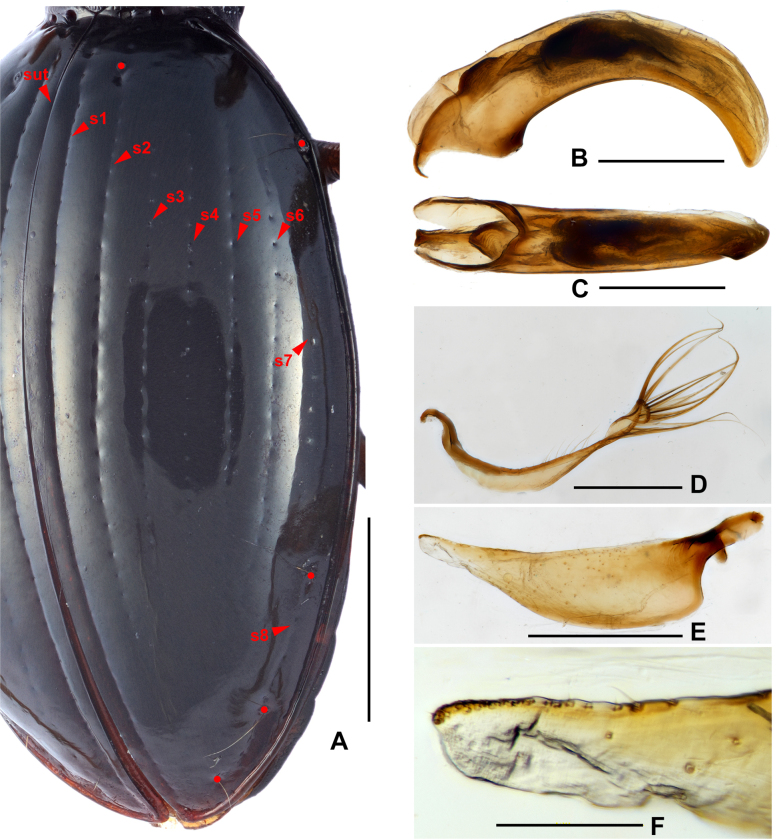
Diagnostic features of *Broscosoma epanggong* sp. nov., male. **A**. Elytra, oblique right lateral view; **B**. Median lobe of aedeagus, lateral view; **C**. Ditto, ventral view; **D**. Right paramere; **E**. Left paramere; **F**. Ditto, apical part. Scale bars: 1 mm (**A**); 0.5 mm (**B–E**); 0.1 mm (**F**). Features in red: sut, elytral suture; s1–s8, elytral striae 1–8. Red dots: setigerous punctures.

***Elytra*** (Fig. 4A) ovoid, strongly convex; shoulders narrow and rounded; each elytron with eight distinct striae, all striae finely punctate, stria 1 deeply incised, striae 2–6 distinct but shallow, striae 7 and 8 very short and shallow, striae 2–8 disappear at the apical portion of the elytra; one parascutellar seta present at base of stria 2, discal setae absent, umbilicate series comprised of one humeral, one preapical and two apical pores.

***Legs*** simple, glabrous, only tibial apex with a few thin setae, protarsomeres 1–4 with adhesive setae ventrally.

***Median lobe of aedeagus*** (Fig. 4B, C) moderately slender, strongly curved and ended in a rounded tip in lateral view, slender and very weakly curved in ventral view; left paramere (Fig. 4E) expanded at middle, apical portion (Fig. 4F) narrowed, with several very short setae at apex of anterior margin, apex rounded; right paramere (Fig. 4D) slender and strongly curved, with dense, long setae along apico-anterior margin.

**Female**: unknown.

#### Measurements

**(mm)**. Males: BL 6.43–7.12; HL 0.97–1.09, HW 1.25–1.36; PL 1.99–2.14, PW 1.62–1.72; EL 3.48–3.93, EW 2.33–2.64.

#### Comparison.

Members of this new species are similar to *B. xumaozhoui* sp. nov. and *B. valainisi*; and they can be easily distinguished from those of the other congeners by the combination of following characters: 1) pronotum and elytra long-oval shaped; 2) pronotal base coarsely punctate and sparsely wrinkled; 3) elytral striae finely punctate, shallow, only stria 1 deeply incised. Compared with *B. valainisi* members, those of this new species can be distinguished by: 1) femora and tibiae yellowish brown (vs dark brown to black in *B. valainisi*); 2) smaller body size, BL < 7.5 mm (BL > 10.0 mm in *B. valainisi*); 3) left paramere with apical portion wider. Compared with *B. xumaozhoui* sp. nov. members, those of *B. epanggong* sp. nov. can be distinguished by: 1) femora lighter in color (reddish to dark brown in *B. xumaozhoui* sp. nov.); 2) shoulders wider (more sloped in *B. xumaozhoui* sp. nov.); 3) right paramere strongly curved (vs slightly curved in *B. xumaozhoui* sp. nov.); 4) apex of left paramere with only several very short setae (vs setae quite longer in *B. xumaozhoui* sp. nov.).

The ranges of *B. xuhaoi* and *B. qiului* are also geographically close to that of *B. epanggong* sp. nov., but *B. xuhaoi* differ from the new species in having: 1) pronotal base smooth, without punctures or wrinkles; 2) elytral striae deeper; 3) legs dark brown; 4) the form of aedeagus different. *Broscosoma qiului* is also differ from the new species in having: 1) pronotum and elytra rounder and shorter; 2) pronotal base usually smooth; 3) the form of aedeagus different.

#### Distribution.

China: only known from the type locality, Mts. Guangwushan in the northeastern part of Sichuan Province.

#### Biological notes.

All adults were collected on the relatively low area of a damp cliff at night (Fig. 16A, B).

#### Etymology.

The specific name *epanggong* is the name of an unfinished palace during the reign of Qin Shi Huang (First emperor of Qin, 259–210 BC). The legend tells that Qin Shi Huang once sourced timber from Guangwushan (the type locality of this new species) to construct the Epang Palace. The name is treated as a noun in apposition.

### Broscosoma jiangyuxini sp. nov.

Taxon classificationAnimaliaColeopteraCarabidae

16D386C2-7E59-5C59-81CA-AE42E0C28253

https://zoobank.org/901921CF-A3F6-4646-8F7C-B64EB56705F2

Figs 5, 6

#### Common name.

姜氏珠步甲.

#### Type material

(1 ♂): ***Holotype***: China: • ♂, labeled ‘China: Guizhou Province (贵州省), Qiannan Buyi and Miao Autonomous Prefecture (黔南布依族苗族自治州), Libo County (荔波县), Maolan Town (茂兰镇), Near the entrance of an unnamed karst cave, H: 655 m, 14.II.2023, Yu-Xin Jiang leg.’ (GUGC).

#### Diagnosis.

Size small, body pyriform; dorsal surface shiny without metallic luster, dark brown to black; legs (except femora), labrum, palpi and antennae brown, femora dark brown; tempora distinctly swollen. Elytra with eight distinct striae, all of them distinctly incised and finely punctate; shoulders largely rounded. Anterior area of pronotum with fine and sparse punctures; basal potion finely wrinkled. Apical part of left paramere strongly narrowed, with apex sharp, anterior margin with several short setae on apical 1/5.

#### Description.

**Figure 5.**
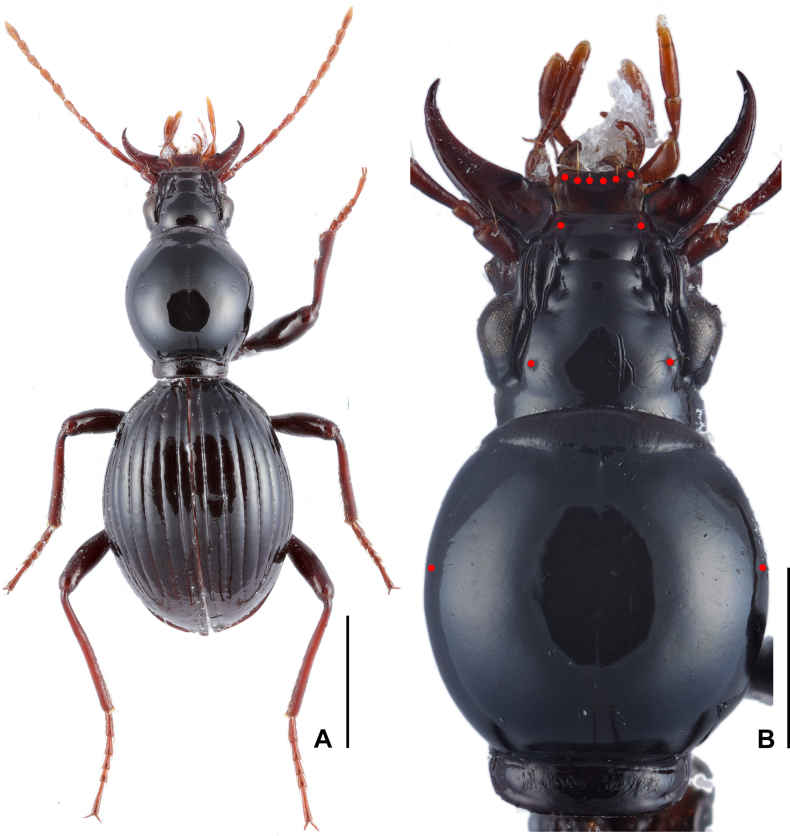
**A**. Dorsal habitus of *Broscosoma jiangyuxini* sp. nov., male; **B**. Ditto, head and pronotum, dorsal view. Scale bars: 2 mm (**A**); 1 mm (**B**). Red dots: setigerous punctures.

**Male. *Body*** (Fig. 5A) pyriform, dorsal surface dark brown to black, shiny, without metallic luster; legs (except femora), labrum, palpi and antennae brown, femora dark brown.

***Head*** (Fig. 5B) finely convex; dorsal surface shiny and smooth, with fine micro-punctures; labrum nearly rectangular, with six setae and concave at apical margin; frontal groove irregular and impunctate; apical margin of clypeus faintly concave with one seta on each side; eyes medium-sized; tempora distinctly swollen.

***Pronotum*** (Fig. 5B) globose, distinctly convex, slightly longer than wide, with maximum width slightly anterior to the middle, equally constricted at anterior and basal margins; dorsal surface with sparse micro-punctures; anterior area of pronotum punctate finely and sparsely; median longitudinal impression and anterior transverse impression shallow but visible; one mid-lateral seta present at anterior middle of pronotum on each side, basal-lateral seta absent; basal portion wrinkled finely, without punctures.

**Figure 6.**
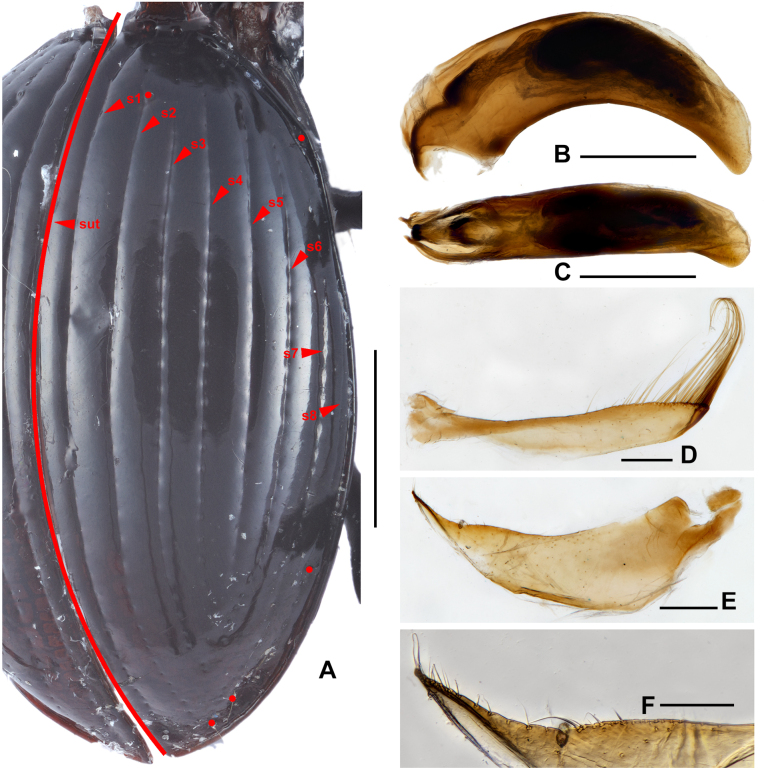
Diagnostic features of *Broscosoma epanggong* sp. nov., male. **A**. Elytra, oblique right lateral view; **B**. Median lobe of aedeagus, lateral view; **C**. Ditto, ventral view; **D**. Right paramere; **E**. Left paramere; **F**. Ditto, apical part. Scale bars: 1 mm (**A**); 0.5 mm (**B, C**); 0.2 mm (**D, E**); 0.1 mm (**F**). Features in red: sut, elytral suture; s1–s8, elytral striae 1–8. Red dots: setigerous punctures.

***Elytra*** (Fig. 6A) ovoid, strongly convex; shoulders extremely narrow and rounded; each elytron with eight distinct striae, all striae distinctly incised and finely punctate, stria 8 relatively shallower; one parascutellar seta present at base of stria 2, discal setae absent, umbilicate series comprised of one humeral, one preapical and two apical pores.

***Legs*** simple, glabrous, only tibial apex with a few thin setae, protarsomeres 1–4 with adhesive setae ventrally.

***Median lobe of aedeagus*** (Fig. 6B, C) simple, curved and ending in a broadly rounded tip in lateral view; slender, nearly straight in basal portion and curved apically in lateral view; left paramere (Fig. 6E) expanded at middle, apical part markedly narrowed, with apex sharp, anterior margin with several short setae on apical fifth; right paramere (Fig. 6D) slender and faintly curved, with dense long setae along apico-anterior margin.

**Female**: unknown.

#### Measurements

**(mm)**. Male: BL 7.17; HL 0.96, HW 1.36; PL 2.24, PW 1.92; EL 3.97, EW 2.90.

#### Comparison.

Members of this new species are most similar to those of *B. zhouhanpingi* sp. nov. in having: 1) small size and pyriform body; 2) dorsal surface without metallic luster; 3) each elytron with eight distinct striae, all striae distinctly incised and finely punctate. However, it can be easily distinguished from the latter by the combination of the following characters: 1) tempora strongly swollen (just slightly swollen in *B. zhouhanpingi* sp. nov.); 2) basal portion of pronotum wrinkled (vs not wrinkled in *B. zhouhanpingi* sp. nov.); 3) elytra dark brown to black (vs brown in *B. zhouhanpingi* sp. nov.); 4) legs with darker color; 5) left paramere apically pointed (vs apex rounded in *B. zhouhanpingi* sp. nov.); 6) median lobe of aedeagus with a rounded tip (vs nearly truncate in *B. zhouhanpingi* sp. nov.).

Members of the new species resemble those of *B. chaoshan* sp. nov. and *B. zhengyuandongi* in their relatively short pyriform body shape. Apart from the comparisons with the latter two species provided under the description of *B. chaoshan* sp. nov., *B. jiangyuxini* sp. nov. members also differ in having: 1) dark brown femora; 2) tempora more swollen; 3) anterior area of pronotum with punctures fine and sparse; 4) elytral stria 8 relatively shallower.

#### Distribution.

China: only known from the type locality, Maolan Town in Guizhou Province.

#### Biological notes.

The only known specimen of this species was collected near the entrance of an unnamed karst cave in the type locality. The type locality is renowned for its well-developed karst landforms, with a high coverage of local karst forest and a humid subtropical monsoon climate.

#### Etymology.

This specific name honors Mr. Yu-Xin Jiang (Harbin City, Heilongjiang Province, China), the collector of the holotype of this new species.

### Broscosoma luojingxiangi sp. nov.

Taxon classificationAnimaliaColeopteraCarabidae

F7E07E55-39DC-535D-B62A-6F66C5C74B78

https://zoobank.org/89F94E5A-F825-4793-BA1E-B818DFBE6296

Figs 7, 8, 17 E

#### Common name.

罗氏珠步甲.

#### Type material

(4 ♀♀): ***Holotype***: China: • ♀, labeled ‘China: Zhejiang Province (浙江省), Lishui City (丽水市), Qingtian County (青田县), near Shigu Lake (师姑湖), H: 1300 m, 02.VI.2024, Jing-Xiang Luo leg.’ (GUGC). ***Paratypes***: • 3 ♀♀, with the same label data as the holotype (2 ♀♀, GUGC; 1 ♀, CBFU).

#### Diagnosis.

Size medium, body elongate-pyriform; dorsal surface shiny without metallic luster, dark brown to black; labrum, palpi and antennae brown to dark brown; legs dark brown to black. Surface of head with relatively distinct micro-punctures. Basal portion of pronotum sparsely punctate, without wrinkles. Elytra with eight distinct striae, all of them slightly incised and coarsely punctate, shoulders narrow and rounded. Gonocoxite I of ovipositor with eight long ensiform setae at dorsal surface and four short ensiform setae at ventral surface; gonocoxite II without setae, distinctly sinuate at basal 1/4 of inner side, apex slightly curved, with a truncated tip.

#### Description.

**Figure 7.**
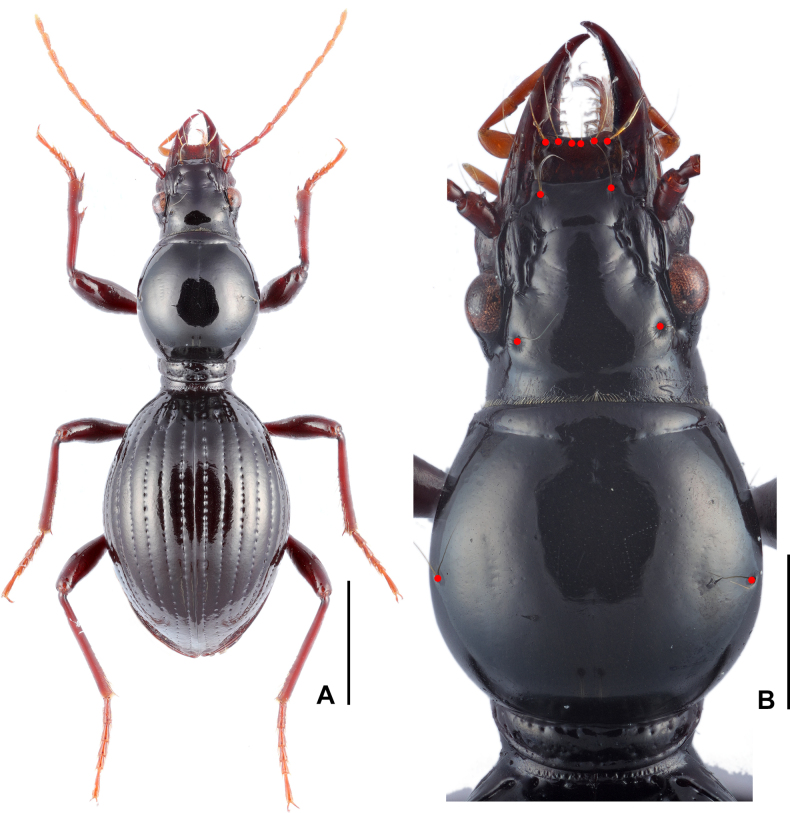
**A**. Dorsal habitus of *Broscosoma luojingxiangi* sp. nov., female; **B**. Ditto, head and pronotum, dorsal view. Scale bars: 2 mm (**A**); 1 mm (**B**). Red dots: setigerous punctures.

**Female. *Body*** (Fig. 7A) elongate-pyriform, dorsal surface dark brown to black, shiny, without metallic luster; legs dark brown to black; labrum, palpi and antennae brown to dark brown.

***Head*** (Fig. 7B) finely convex; dorsal surface shiny and smooth, with relatively distinct and dense micro-punctures; labrum nearly rectangular, with six setae and apical margin slightly concave; frontal grooves irregular and impunctate; apical margin of clypeus faintly convex, with one seta on each side; eyes medium-sized; tempora slightly swollen.

***Pronotum*** (Fig. 7B) globose, distinctly convex, slightly longer than wide, with maximum width slightly anterior to the middle, equally constricted at anterior and basal margins; dorsal surface with sparse micro-punctures; median longitudinal impression and anterior transverse impression shallow but distinct; one mid-lateral seta present at anterior middle of pronotum on each side, basal-lateral seta absent; basal portion sparsely and coarsely punctate, without wrinkles.

**Figure 8.**
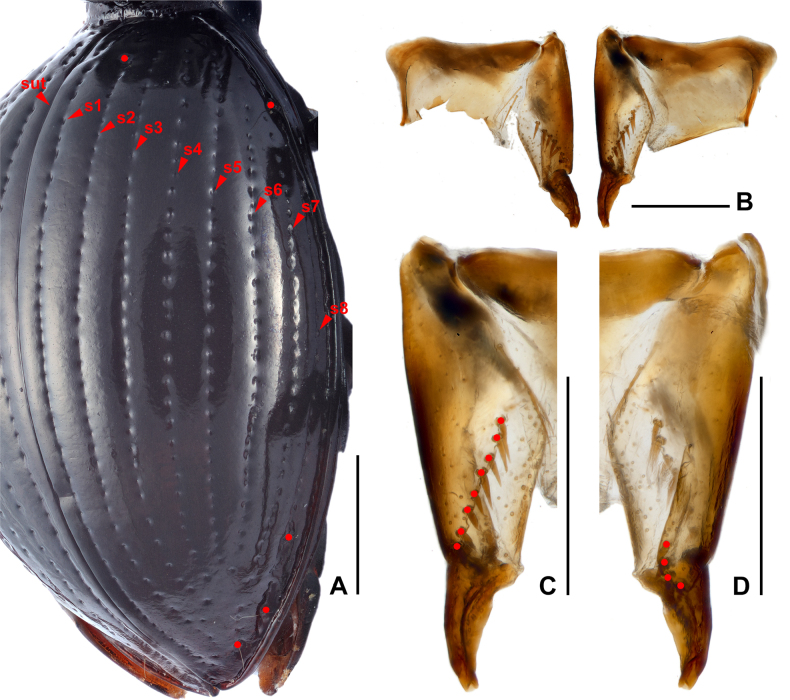
Diagnostic features of *Broscosoma luojingxiangi* sp. nov., female. **A**. Elytra, oblique right lateral view; **B**. Left and right ovipositor, dorsal view; **C**. Right ovipositor, dorsal view; **D**. Ditto, ventral view. Scale bars: 1 mm (**A**); 0.5 mm (**B–D**). Features in red: sut, elytral suture; s1–s8, elytral striae 1–8. Red dots: setigerous punctures (**A**); setae and setigerous punctures (**C, D**).

***Elytra*** (Fig. 8A) ovoid, strongly convex, dorsal surface shiny; shoulders narrow and rounded; each elytron with eight distinct striae, all striae slightly incised and coarsely punctate; one parascutellar seta present at base of stria 2, discal setae absent, umbilicate series comprised of one humeral, one preapical and two apical pores.

***Legs*** simple, glabrous, only tibial apex with a few thin setae, protarsomeres 1–4 without adhesive setae ventrally.

***Ovipositor*** (Fig. 8B–D), gonocoxite I of ovipositor with eight long ensiform setae at dorsal surface (Fig. 8C) and four short ensiform setae at ventral surface (Fig. 8D); gonocoxite II without setae, distinctly sinuate at basal 1/4 of inner side, apex slightly curved, with a truncated tip (Fig. 8C, D).

**Male**: Unknown.

#### Measurements

**(mm)**. Females: BL 8.78–9.31; HL 1.19–1.23, HW 1.60–1.63; PL 2.67–2.79, PW 2.16–2.40; EL 4.92–5.29, EW 3.33–3.57.

#### Comparison.

Members of this new species are most similar to those of *B. wuyishanum* and *B. surongxiangi* sp. nov., all of which have adjacent distribution ranges, an elongate-pyriform body shape, dark colored legs, and distinct, relatively dense micro-punctures on the head and pronotum.

Members of this new species are quite similar to those of *B. wuyishanum* in appearance, however, those of the new species can be distinguished from those of the latter by the differences of gonocoxite 2 of ovipositor as following: 1) without seta (vs with a nematiform seta in *B. wuyishanum* females); 2) nearly straight, just slightly curved near apex (vs distinctly curved from base to apex in *B. wuyishanum* females); 3) tip truncate (vs tip narrowly rounded in *B. wuyishanum* females); 4) distinctly sinuate at inner side of basal 1/4 (vs smoothly curved in *B. wuyishanum* females). Compared with females of *B. surongxiangi* sp. nov. and *B. luojingxiangi* sp. nov. differ in having: 1) body size larger, BL > 8.60 mm (vs BL 8.27 mm in *B. surongxiangi* sp. nov.); 2) pronotal base with punctures denser; 3) elytral striae 3–8 incised slightly but distinctly (vs shallow in *B. surongxiangi* sp. nov.); 4) punctures of elytral striae coarser.

#### Distribution.

China: only known from the type locality, Qingtian County in Zhejiang Province.

#### Biological notes.

Females of this species were collected on the relatively low area of damp cliff at night (Fig. 17E).

#### Etymology.

This specific name honors Mr. Jing-Xiang Luo (Lishui City, Zhejiang Province, China), the collector of the type series of this new species.

### Broscosoma surongxiangi sp. nov.

Taxon classificationAnimaliaColeopteraCarabidae

6EA8182C-C78F-5D70-9DAA-A61147E6D114

https://zoobank.org/3C6F015D-1465-4F97-A49E-D09FAB0B7ED9

Figs 9, 10, 17 A

#### Common name.

苏氏珠步甲.

#### Type material

(1 ♂): ***Holotype***: China: • ♂, labeled ‘China: Fujian Province (福建省), Wuyishan City (武夷山市), Yangzhuang Twonship (洋庄乡), Huanggang Shan (黄岗山), H: 1815–1820 m, 30.VI.2024, Rong-Xiang Su, Zhi-Hao Qi & Zhi-Ning Liao leg.’ (GUGC).

#### Diagnosis.

Size medium, body elongate-pyriform; dorsal surface shiny without metallic luster, dark brown to black; labrum, palpi and antennae brown to dark brown; legs dark brown to black. Surface of head with relatively distinct micro-punctures. Basal portion of pronotum sparsely punctate, without wrinkles. Elytra with striae 1 distinctly incised, striae 2–8 shallow, stria 8 almost effaced; shoulders narrow and rounded. Aedeagus strongly curved and ending in a short, truncated tip; apical part of left paramere covered with a row of very short setae along the anterior margin.

#### Description.

**Figure 9.**
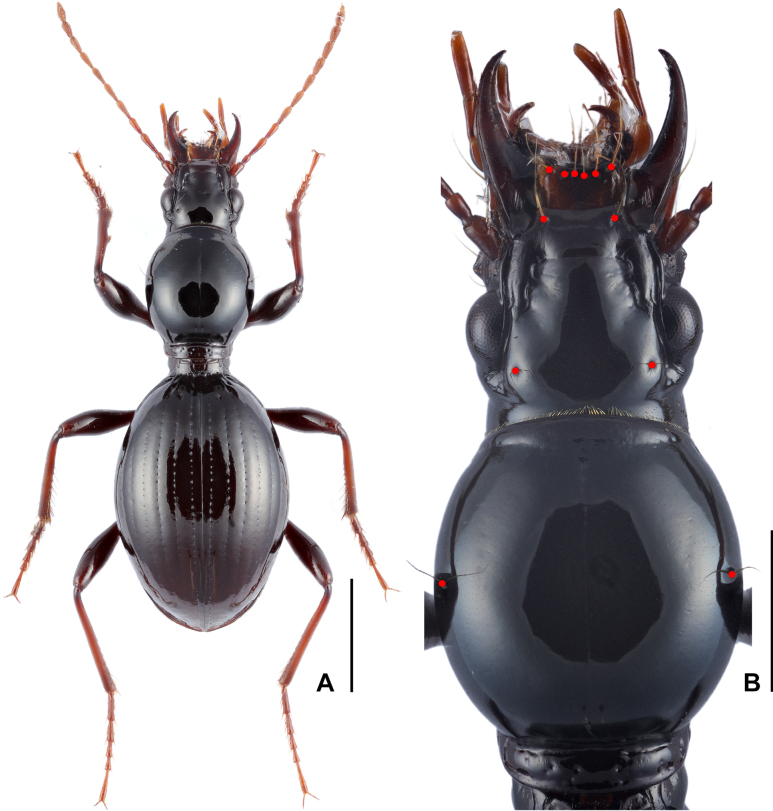
**A**. Dorsal habitus of *Broscosoma surongxiangi* sp. nov., male; **B**. Ditto, head and pronotum, dorsal view. Scale bars: 2 mm (**A**); 1 mm (**B**). Red dots: setigerous punctures.

**Male. *Body*** (Fig. 9A) elongate-pyriform, dorsal surface dark brown to black, shiny, without metallic luster; legs dark brown to black; labrum, palpi and antennae brown to dark brown.

***Head*** (Fig. 9B) finely convex; dorsal surface shiny and smooth, with relatively distinct and dense micro-punctures; labrum nearly rectangular, with six setae and apical margin slightly concave; frontal grooves irregular and impunctate; apical margin of clypeus slightly concave with one seta on each side; eyes medium-sized; tempora slightly swollen.

***Pronotum*** (Fig. 9B) globose, distinctly convex, longer than wide, with maximum width slightly anterior to the middle, equally constricted at anterior and basal margins; dorsal surface with sparse micro-punctures; median longitudinal impression and anterior transverse impression shallow but distinct; one mid-lateral seta present at anterior middle of pronotum on each side, basal-lateral seta absent; basal portion sparsely and coarsely punctate, without wrinkles.

**Figure 10.**
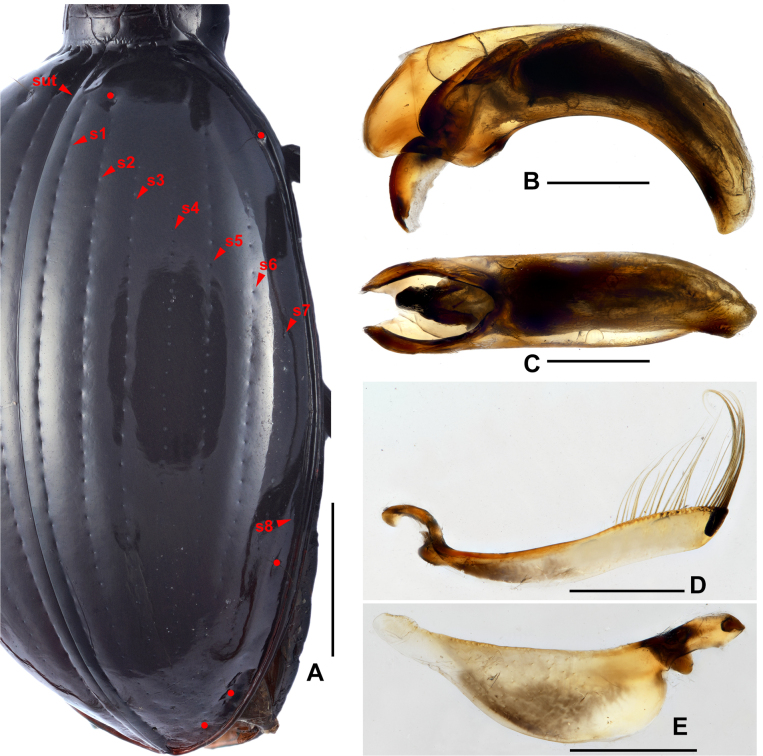
Diagnostic features of *Broscosoma surongxiangi* sp. nov., male. **A**. Elytra, oblique right lateral view; **B**. Median lobe of aedeagus, lateral view; **C**. Ditto, ventral view; **D**. Right paramere; **E**. Left paramere. Scale bars: 1 mm (**A**); 0.5 mm (**B–E**). Features in red: sut, elytral suture; s1–s8, elytral striae 1–8. Red dots: setigerous punctures.

***Elytra*** (Fig. 10A) ovoid, strongly convex; shoulders narrow and rounded; each elytron with eight striae, striae 1 distinctly incised, striae 2–8 shallow, stria 8 almost invisible, with only a shallow impression remaining, all striae finely punctate; one parascutellar seta present at base of stria 2, discal setae absent, umbilicate series comprised of one humeral, one preapical and two apical pores.

***Legs*** simple, glabrous, only tibial apex with a few thin setae, protarsomeres 1–4 with adhesive setae ventrally.

***Median lobe of aedeagus*** (Fig. 10B, C) simple, curved and ended in a truncated tip in lateral view, slender and weakly curved apically in ventral view. Left paramere (Fig. 10E) expanded at middle, apex with a row of very short setae along the anterior margin; right paramere (Fig. 10D) slender, curved, with dense long setae along apico-anterior margin.

**Female**: Unknown.

#### Measurements

**(mm)**. Male: BL 8.27; HL 1.19, HW 1.46; PL 2.41, PW 1.94; EL 4.67, EW 3.03.

#### Comparison.

Members of this new species can be confused with those of its sympatric species *B. wuyishanum*, but can be readily distinguished from them by the following characters: 1) body size smaller, BL 8.27 mm (vs BL > 9.0 mm in *B. wuyishanum*); 2) elytral striae shallower, stria 8 almost visible (vs elytral striae deeper, and stria 8 normal in *B. wuyishanum*); 3) punctures of elytral striae finer (distinctly coarser in *B. wuyishanum*); 4) median lobe of aedeagus less curved, with apex narrower (vs strongly curved, with apex widely rounded in *B. wuyishanum*); 5) apex of left paramere wider.

Members of this new species are also similar to those of *B. luojingxiangi* sp. nov. The differences between *B. surongxiangi* sp. nov. and *B. luojingxiangi* sp. nov. members are noted in the comparison section for *B. luojingxiangi* sp. nov.

#### Distribution.

China: only known from the type locality, Huanggang Mountain of Wuyishan City in Fujian Province.

#### Biological notes.

The holotype male of this new species was collected under a roadside stone (Fig. 17A).

#### Etymology.

This specific name honors Mr. Rong-Xiang Su (Fuzhou City, Fujian Province, China), one of the collectors of the holotype of this new species.

### Broscosoma xumaozhoui sp. nov.

Taxon classificationAnimaliaColeopteraCarabidae

2BC7CE01-77AB-5FED-9DCD-04028553BC6E

https://zoobank.org/E46EADBC-139D-44BD-BFB6-98D00E469C86

Figs 11, 12, 17 B–D

#### Common name.

徐氏珠步甲.

#### Type material

(5 ♂♂, 1 ♀): ***Holotype***: China: • ♂, labeled ‘China: Hubei Province (湖北省), Shennongjia Forestry District (神农架林区), Muyu Town (木鱼镇), Shennongding Scenic Spot (神农顶景区), Jinhouling (金猴岭), H: ~2400 m, 23.VI.2025, Mao-Zhou Xu leg.’ (GUGC). ***Paratypes***: • 4 ♂♂, with the same label data as the holotype (GUGC); 1 ♀, labeled ‘Chongqing (重庆), Wuxi (巫溪), Yintiaoling (阴条岭), Guanshan Forest Centre (官山林场), Shizhuzi (石柱子), 2137 m, 31.53750°N, 109.69711°E, Fagaceae forest; 2024.VIII.11; Jiaheng Chen & Luyu Wang lgt.’ (CBFU).

#### Diagnosis.

Size small, body elongate-pyriform; dorsal surface shiny with extremely weak metallic luster, dark brown to black; palpi, antennae, labrum, mandibles and femora reddish brown, tibiae and tarsi yellowish brown. Elytral stria 1 deeply incised, striae 2–7 distinct but shallow, striae 6 and 7 shallower than 2–5, stria 8 very short and shallow, almost effaced, with only a shallow impression remaining; shoulders largely rounded. Basal portion of pronotum sparsely and coarsely punctate, with just a few punctures connected forming short wrinkles. Aedeagus strongly curved and ending in a rounded tip, apical part of left paramere covered with a row of setae in different lengths along the anterior margin, some of them much longer than others.

#### Description.

**Figure 11.**
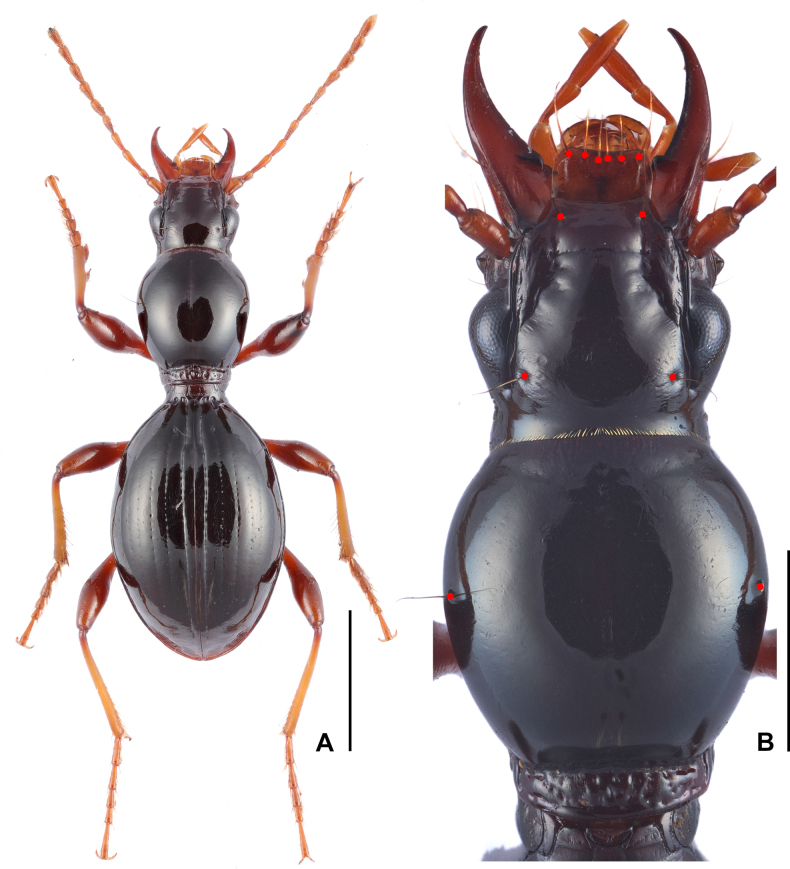
**A**. Dorsal habitus of *Broscosoma xumaozhoui* sp. nov., male; **B**. Ditto, head and pronotum, dorsal view. Scale bars: 2 mm (**A**); 1 mm (**B**). Red dots: setigerous punctures.

**Male. *Body*** (Fig. 11A) elongate-pyriform, dorsal surface dark brown to black, much shiny, with extremely weak metallic luster; palpi, antennae, labrum, and mandibles reddish brown; femora reddish brown, tibiae and tarsi yellowish brown.

***Head*** (Fig. 11B), finely convex; dorsal surface shiny, with sparse micro-punctures; labrum nearly rectangular, with six setae and apical margin slightly concave; frontal grooves irregular and impunctate; apical margin of clypeus moderately concave, with one seta on each side; eyes medium-sized, moderately convex.

***Pronotum*** (Fig. 11B) globose, distinctly convex, longer than wide, with maximum width anterior to the middle, equally constricted at anterior and basal margins; dorsal surface with sparse micro-punctures; median longitudinal impression and anterior transverse impression shallow but distinct; one mid-lateral seta present at anterior middle of pronotum on each side, basal-lateral seta absent; basal portion sparsely and coarsely punctate.

**Figure 12.**
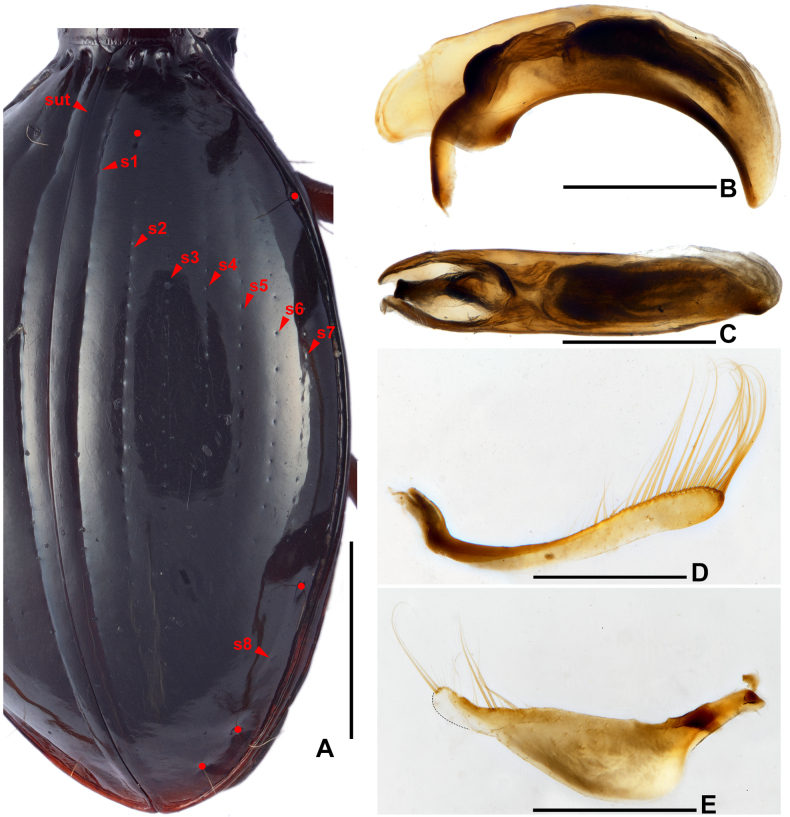
Diagnostic features of *Broscosoma xumaozhoui* sp. nov., male. **A**. Elytra, oblique right view; **B**. Median lobe of aedeagus, lateral view; **C**. Ditto, ventral view; **D**. Right paramere; **E**. Left paramere. Scale bars: 1 mm (**A**); 0.5 mm (**B–E**). Features in red: sut, elytral suture; s1–s8, elytral striae 1–8. Red dots: setigerous punctures.

***Elytra*** (Fig. 12A) ovoid, strongly convex; shoulders narrow and rounded; each elytron with eight striae, stria 1 deeply incised, striae 2–7 distinct but shallow, striae 6 and 7 shallower than 2–5, stria 8 very short and shallow, almost effaced, with only a shallow impression remaining, all striae finely punctate, not extended to the anterior and posterior margins of elytra; one parascutellar seta present at base of stria 1, discal setae absent, umbilicate series comprised of one humeral, one preapical and two apical pores.

***Legs*** simple, glabrous, only tibial apex with a few thin setae, protarsomeres 1–4 with adhesive setae ventrally.

***Median lobe of aedeagus*** (Fig. 12B, C) strongly curved and ended in a narrowly rounded tip in lateral view, slender and apical half weakly curved in ventral view; left paramere (Fig. 12E) weakly expanded at middle, apical portion narrowed ending in a widely rounded tip, with a row of long setae in different lengths along the anterior margin, some of them much longer than others; right paramere (Fig. 12D) slender and distinctly curved at basal 1/2, with dense long setae along apico-anterior margin.

**Female**: externally similar to the male, protarsomeres 1–4 without adhesive setae ventrally.

#### Measurements

**(mm)**. Males: BL 6.28–6.81; HL 0.94–1.06, HW 1.23–1.33; PL 1.81–2.03, PW 1.45–1.64; EL 3.53–3.89, EW 2.39–2.55. Females: BL 7.10; HL 1.25, HW 1.33; PL 2.00, PW 1.71; EL 3.85, EW 2.65.

#### Comparison.

Members of this new species are very similar to those of *B. epanggong* sp. nov. and *B. valainisi*. The differences between members of these three species and other congeners, and the differences between *B. xumaozhoui* sp. nov. and *B. epanggong* sp. nov., are discussed in the comparison section for *B. epanggong* sp. nov.

Members of *B. xumaozhoui* sp. nov. and *B. valainisi* can be distinguished as follows: 1) body size smaller, BL < 7.0 mm (vs BL > 10.0 mm in *B. valainisi*); 2) elytral shoulder narrower; 3) elytra form shorter, EL/EW < 1.6 (EL/EW > 1.6 in *B. valainisi*); 4) left paramere with apex wider, ended in a widely rounded tip (ended in a narrow tip in *B. valainisi*); 5) left paramere with long setae along the anterior margin (vs without setae in *B. valainisi*).

#### Distribution.

China: only known from two adjacent localities: Shennongjia in western part of Hubei Province and Yintiaoling N. R. in eastern part of Chongqing Municipality.

#### Biological notes.

**Figure 13.**
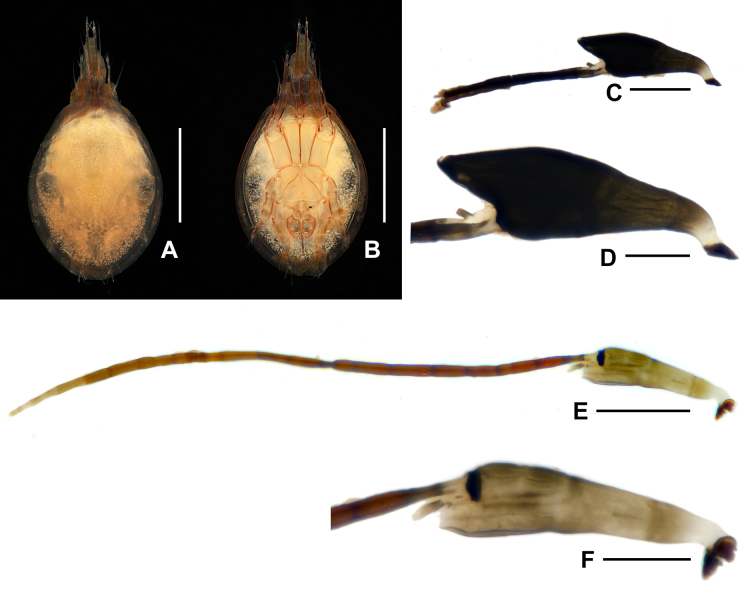
Attachments on the body surface of *Broscosoma xumaozhoui* sp. nov. **A**. Hypopodes of Acaridae, dorsal view; **B**. Ditto, ventral view; **C, E**. Laboulbeniomycetes, habitus; **D, F**. Ditto, basal part. Scale bars: 0.2 mm (**A, B**); 0.1 mm (**C, E**); 0.05 mm (**D, F**). **A, B**. Taken by Mr. Bao-Xiang Zhan.

All adults from Hubei were collected on the lower area of a damp cliff at night (Fig. 17B–D). The paratype from Chongqing was collected on wooden trestle in a Fagaceae forest. The body surface of the specimens from Hubei Province carried hypopodes of Acaridae (Fig. 13A, B) and Laboulbeniomycetes (Fig. 13C–F).

#### Etymology.

This specific name honors Mr. Mao-Zhou Xu (Yantai City, Shandong Province, China), one of the collectors of specimens of this new species.

### Broscosoma zhouhanpingi sp. nov.

Taxon classificationAnimaliaColeopteraCarabidae

3F8547EB-AC9A-5507-8EE7-04C6FEECA927

https://zoobank.org/536FE16B-F23C-41DE-A605-7B66A543E336

Figs 14, 15, 18

#### Common name.

周氏珠步甲.

#### Type material

(1 ♂): ***Holotype***: China: • ♂, labeled ‘China: Chongqing City (重庆市), Qijiang District (綦江区), Xinwu Village (新五村), Chongqing Qijiang National Geological Park (重庆綦江国家地质公园), Gujianshan Scenic Area (古剑山景区), 28°58'19.35"N, 106°37'23.7"E, H: ~552 m, 19.II.2021, Han-Ping Zhou leg.’ (GUGC).

#### Diagnosis.

Size small, body pyriform; dorsal surface shiny, without metallic luster, head and pronotum (except basal portion) dark brown to black, elytra and basal portion of pronotum reddish brown to dark brown; femora and mandibles reddish brown to dark brown; tibiae and tarsi yellowish brown. Elytra with eight distinct striae, all striae distinctly incised and finely punctate; shoulders largely rounded. Pronotum with micro-punctures on anterior portion slightly denser than on other areas; basal portion faintly punctate in anterior and posterior grooves. Aedeagus with median lobe ending in a truncated tip; left paramere with tip widely rounded, with a row of short setae in different lengths along the anterior margin, setae on apex much longer than that on other parts.

#### Description.

**Figure 14.**
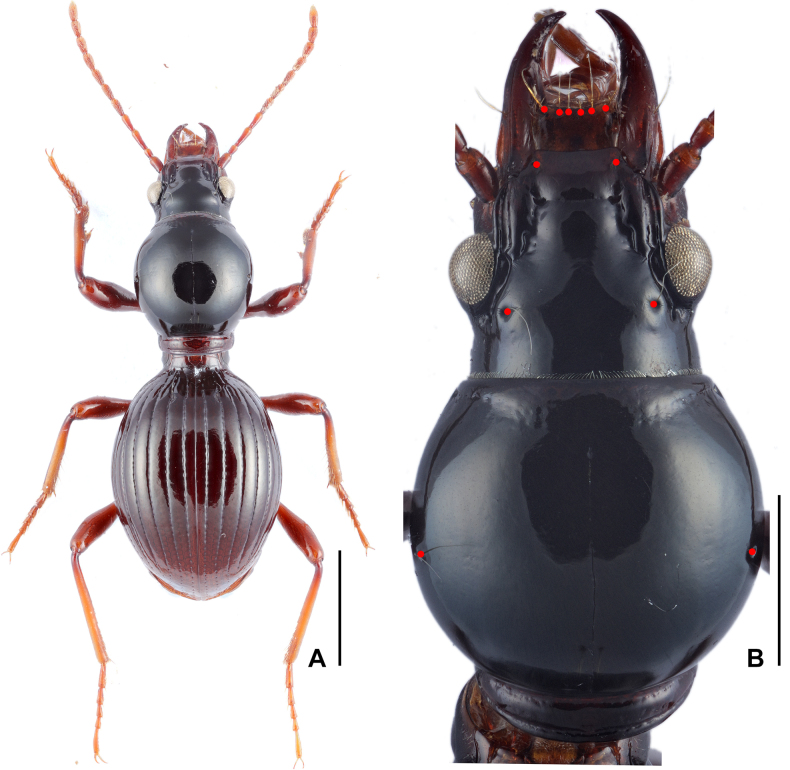
**A**. Dorsal habitus of *Broscosoma zhouhanpingi* sp. nov., male; **B**. Ditto, head and pronotum, dorsal view. Scale bars: 2 mm (**A**); 1 mm (**B**). Red dots: setigerous punctures.

**Male. *Body*** (Fig. 14A) pyriform; dorsal surface shiny, without metallic luster, head and pronotum (except basal portion) dark brown to black, elytra and basal portion of pronotum reddish brown to dark brown; femora and mandibles reddish brown to dark brown; tibiae and tarsi yellowish brown.

***Head*** (Fig. 14B) finely convex; dorsal surface shiny, with micro-punctures sparse and almost indistinct; labrum nearly rectangular, with six setae and apical margin slightly concave; frontal grooves irregular and impunctate; apical margin of clypeus nearly straight with one seta on each side; eyes medium-sized, finely convex; tempora short and swollen.

***Pronotum*** (Fig. 14B) globose, distinctly convex, slightly longer than wide, with maximum width slightly anterior to the middle, equally constricted at anterior and basal margins; dorsal surface shiny and with sparse micro-punctures, micro-punctures near anterior margin slightly denser than that on other area; median longitudinal impression and anterior transverse impression shallow but distinct; one mid-lateral seta present at anterior middle of pronotum on each side, basal-lateral seta absent; basal portion faintly punctate in anterior and posterior grooves.

**Figure 15.**
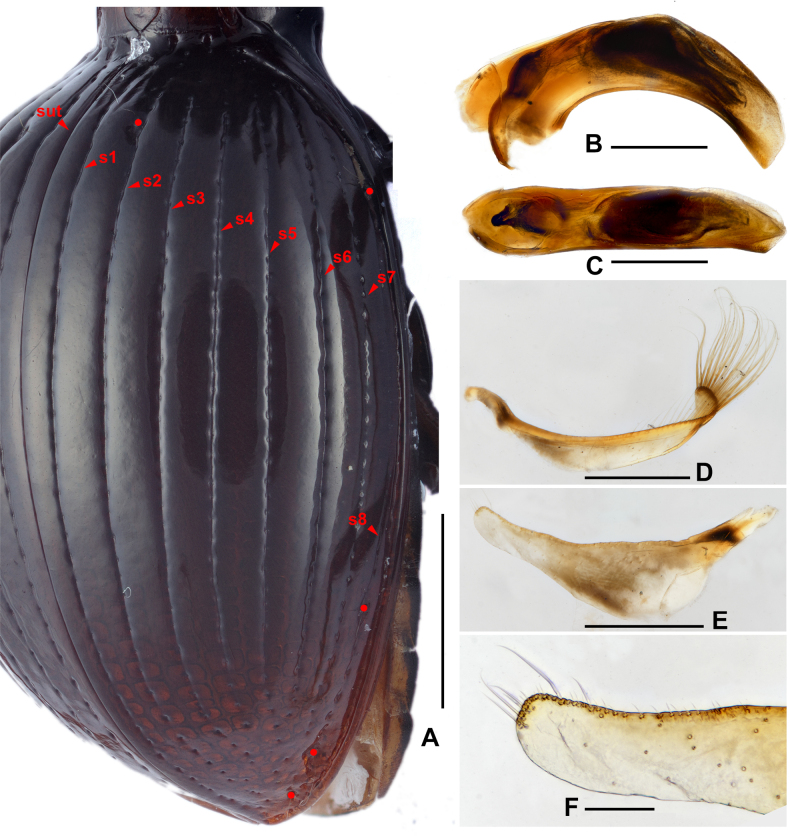
Diagnostic features of *Broscosoma zhouhanpingi* sp. nov., male. **A**. Elytra, oblique right view; **B**. Median lobe of aedeagus, lateral view; **C**. Ditto, ventral view; **D**. Right paramere; **E**. Left paramere; **F**. Ditto, apical part. Scale bars: 1 mm (**A**); 0.5 mm (**B, C**); 0.2 mm (**D, E**); 0.1 mm (**F**). Features in red: sut, elytral suture; s1–s8, elytral striae 1–8. Red dots: setigerous punctures.

***Elytra*** (Fig. 15A) ovoid, strongly convex; shoulders extremely narrow and rounded; each elytron with eight distinct striae, all striae distinctly incised and finely punctate; one parascutellar seta present at base of stria 2, discal setae absent, umbilicate series comprised of one humeral, one preapical and two apical pores.

***Legs*** simple, glabrous, only tibial apex with a few thin setae, protarsomeres 1–4 with adhesive setae ventrally.

***Median lobe of aedeagus*** (Fig. 15B, C) simple, curved and ended in a truncated tip in lateral view; nearly straight and apex weakly curved in ventral view; left paramere (Fig. 15E, F) expanded at middle, apical part narrowed, with apex widely rounded, apical part with several short setae along the anterior margin; right paramere (Fig. 15D) slender and curved, rounded at apex, with dense long setae along apicoanterior margin.

**Figure 16.**
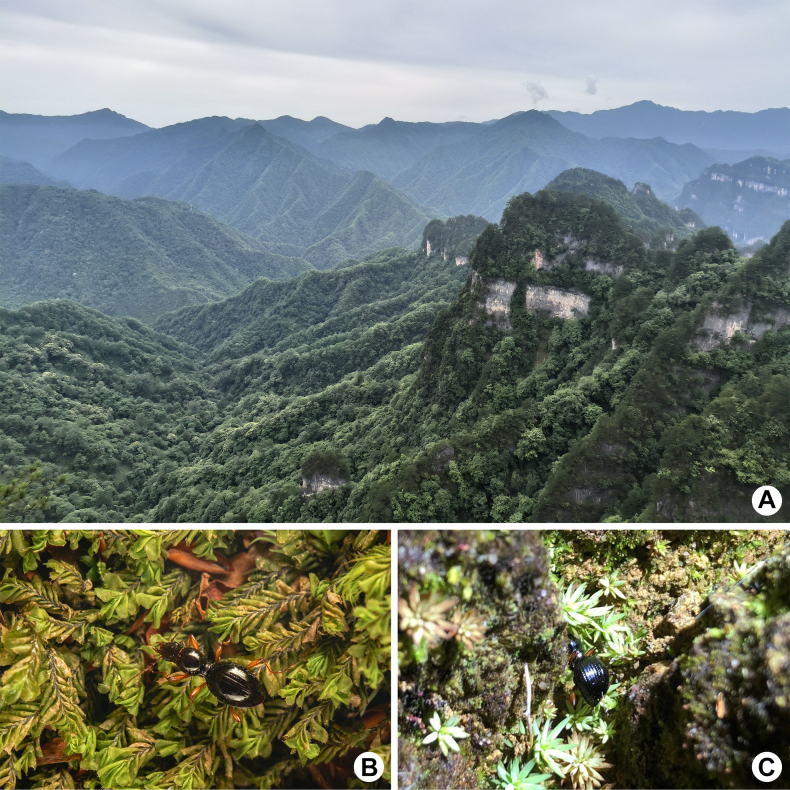
Habitats of *Broscosoma* species. **A**. General environment of Guangwushan Mountain, Sichuan Province, China; **B**. Living adult of *B. epanggong* sp. nov.; **C**. Living adult of *B. chaoshan* sp. nov. **A, B**. Photographs taken by Mr. Mao-Zhou Xu.

**Figure 17.**
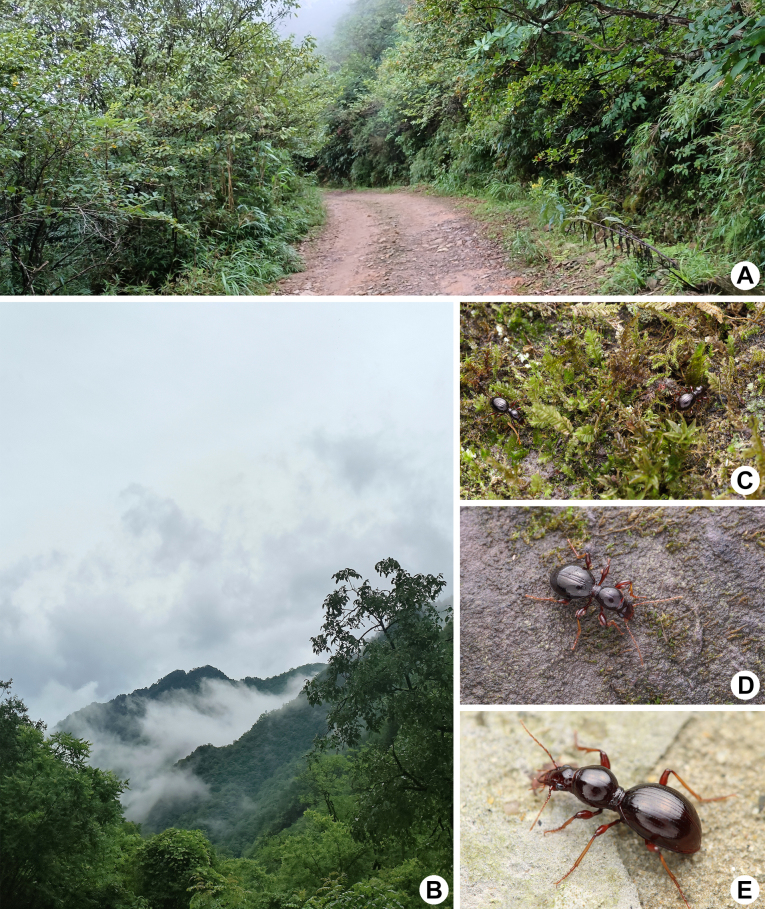
Habitats of *Broscosoma* species. **A**. General environment of Huanggangshan Mountain, Fujian province, China; **B**. General environment of Jinhouling, Hubei Province China; **C, D**. Living adults of *B. xumaozhoui* sp. nov.; **E**. Living adult of *B. luojingxiangi* sp. nov. **A**. Photograph taken by Mr. Xiang-Rong Su; **B–D**. Photographs taken by Mr. Mao-Zhou Xu; **E**. Photograph taken by Mr. Jing-Xiang Luo.

**Figure 18.**
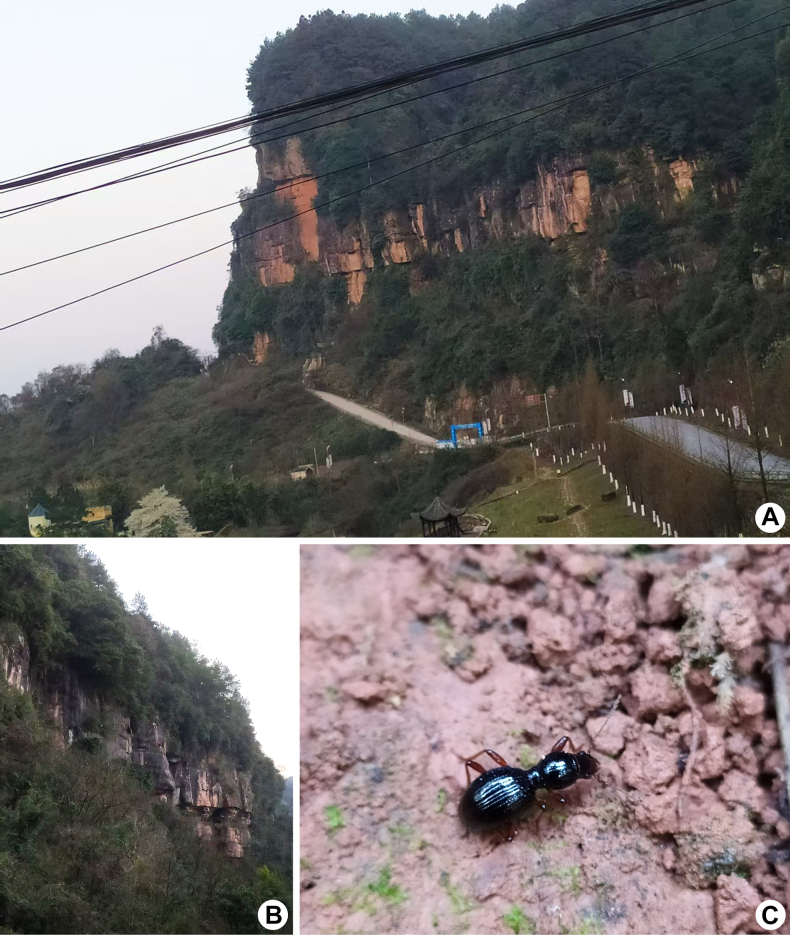
Habitat of *Broscosoma zhouhanpingi* sp. nov. **A, B**. General environment of Gujianshan Scenic Area, Chongqing City, China; **C**. Living adult of *B. zhouhanpingi* sp. nov. All photographs taken by Mr. Han-Ping Zhou.

**Figure 19.**
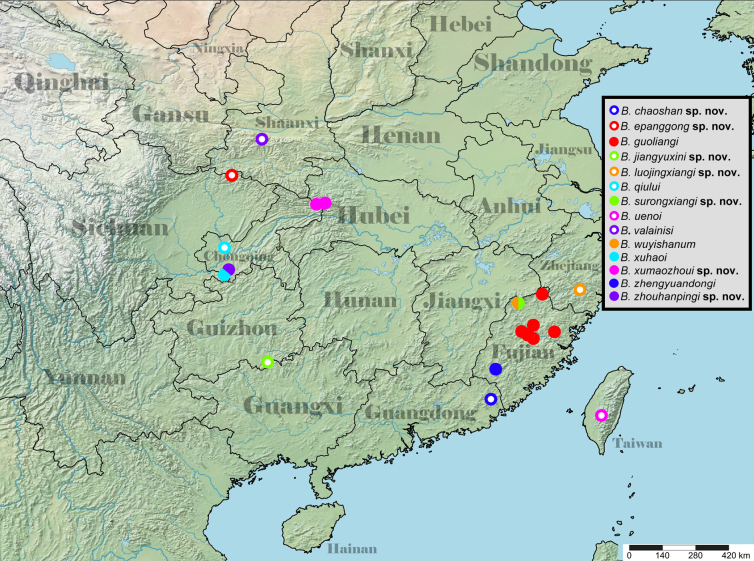
Distribution map of Chinese *Broscosoma* species outside the Qinghai-Xizang Plateau and Hengduan Mountains region.

**Female**: unknown.

#### Measurements

**(mm)**. Male: BL 7.55; HL 1.00, HW 1.52; PL 2.46, PW 2.04; EL 4.09, EW 2.95.

#### Comparison.

Members of this new species are very similar to those of *B. chaoshan* sp. nov., *B. jiangyuxini* sp. nov. and *B. zhengyuandongi*. See the comparison sections for those species above for discussions of distinguishing features.

The geographical range of *B. zhouhanpingi* sp. nov. is also close to those of *B. xuhaoi* and *B. qiului*; but its members can be readily distinguished from these species by: 1) body short-pyriform (elongate-pyriform in *B. xuhaoi*); 2) elytral striae deeply incised (faintly incised in *B. xuhaoi*; shallow in *B. qiului*); 3) shoulders narrow (wider and slightly swollen in *B. qiului*); 4) form of the male aedeagus different.

#### Distribution.

China: only known from the type locality, Chongqing Qijiang National Geological Park in Chongqing City.

#### Biological notes.

The specimen was collected near moist soil at the base of a cliff.

#### Etymology.

This specific name honors Mr. Han-Ping Zhou (Chongqing City, China), the collector of the holotype of this new species.

## Supplementary Material

XML Treatment for Broscosoma chaoshan

XML Treatment for Broscosoma epanggong

XML Treatment for Broscosoma jiangyuxini

XML Treatment for Broscosoma luojingxiangi

XML Treatment for Broscosoma surongxiangi

XML Treatment for Broscosoma xumaozhoui

XML Treatment for Broscosoma zhouhanpingi

## Additional information

### Artificial Intelligence (AI) use

The authors accept full responsibility for the content of the manuscript, including the disclosure of any use of AI.

No AI tools were used in the preparation of this manuscript.

### Author ORCIDs

J.-H. Chen https://orcid.org/0000-0003-2449-8838

X.-S. Chen https://orcid.org/0000-0001-9801-0343

### Data availability

All of the data that support the findings of this study are available in the main text.

## References

[B1] Andrewes HE (1927) Descriptions of some new species of Carabidae from North India. Eos, Revista Espanola de Entomologia 3: 65–77.

[B2] Barševskis A (2010) New species of *Broscosoma* Rosenhauer, 1846 (Coleoptera: Carabidae) from China. Baltic Journal of Coleopterology 10(1): 19–22.

[B3] Bousquet Y (2003) Broscinae. In: Löbl I, Smetana A (Ed.) Catalogue of Palearctic Coleoptera (Vol. 1). Apollo Books, Stenstrup, 235–237.

[B4] Deuve T (1990) Nouveaux Carabidae et Broscidae des montagnes tibeto-himalayennes (Coleoptera). Revue Francaise d’Entomologie, New Series 12: 183–190.

[B5] Deuve T (1992) Un noveau *Broscosoma* du Sichuan (Col., Broscidae). Nouvelle Revue d’Entomologie, New Series 9: e338.

[B6] Deuve T (2006a) Nouveaux *Carabus*, *Cychrus* et *Broscosoma* de Chine (ColeopteraCarabidae et Broscidae). Coléoptères 12(29): 383–398.

[B7] Deuve T (2006b) Nouveaux *Carabus* et *Broscosoma* de Chine (ColeopteraCarabidae et Broscidae). Coléoptères 12(31): 415–425.

[B8] Deuve T (2008a) Deux nouveaux *Broscosoma* Rosenhauer, 1846, du Sichuan (Col., Caraboidea, Broscidae). Bulletin de la Societe Entomologique de France 113(2): 255–256.

[B9] Deuve T (2008b) Nouveaux *Carabus*, *Leistus* et *Broscosoma* de Chine (Coleoptera: Carabidae, Broscidae, Nebriidae). Nouvelle Revue d’Entomologie, New Series 24(3): 259–266.

[B10] Deuve T (2011a) Nouveaux Nebriidae, Broscidae et Trechidae de Chine et d’Iran. Revue Francaise d’Entomologie, New Series 32(1–2): 1–24.

[B11] Deuve T (2011b) Nouveaux *Leistus* et *Broscosoma* de Chine et du Népal (Coleoptera, Caraboidea). Nouvelle Revue d’Entomologie 27(2): 151–161.

[B12] Deuve T (2014) Nouveax *Cychrus*, *Carabus* et *Broscosoma* de Chine occidentale (Coleoptera, Carabidae, Broscidae). Coléoptères 20(9): 65–84.

[B13] Deuve T (2018) Nouveaux *Carabus* L., 1758, et *Broscosoma* Rosenhauer, 1846, de Chine et de Géorgie (Coleoptera, Carabidae et Broscidae). Coléoptères 24(5): 43–50.

[B14] Deuve T (2023) Note sur deux *Carabus* d’Italie et de Géorgie et nouveaux *Leistus*, *Broscosoma* et *Amerizus* de Chine (ColeopteraCaraboidea). Coléoptères 29: 53–64.

[B15] Deuve T, Tian M-Y (2002) Un noveaux *Broscosoma* de la Chine subtropicale (Col., Broscidae). Bulletin de la Societe Entomologique de France 107(4): 395–396. 10.3406/bsef.2002.16880

[B16] Deuve T, Wrase DW (2015) Description d’un nouveau *Broscosoma* du Yunnan, Chine (Coleoptera, Caraboidea, Broscidae). Bulletin de la Societe Entomologique de France 120(1): 29–30. 10.3406/bsef.2015.2201

[B17] Deuve T, Shi H-L, Liang H-B (2025) Descriptions de trois nouveaux *Broscosoma* Rosenhauer, 1846, de Chine et notes sur la présence de ce genre au Tibet (Coleoptera, Caraboidea, Broscinae). Bulletin de la Société entomologique de France 130(2): 167–178. 10.32475/bsef_2377

[B18] Dvořak M (1998) Neue *Broscosoma*-Art aus Tibet (Coleoptera: Carabidae, Broscini). Folia Heyrovskyana 6: 73–75.

[B19] Facchini S (2002) Description of *Broscosoma tibetanum* from Tibet (Coleoptera, Carabidae, Broscinae). Giornale italiano di Entomologia 135: 153–157.

[B20] Habu A (1973) Notes and description of Formosan Carabidae Taken by Dr S-I Ueno in 1961 (Coleoptera, Carabidae). II. A new *Broscosoma* and two new *Patrobus* species. Transactions of Shikoku Entonological Society 11(4): 99–106.

[B21] Häckel M (2017) Broscini Hope, 1838. In: Löbl I, Löbl D (Eds) Catalogue of Palaearctic Coleoptera (Vol. 1). Revised and Updated Edition. Archostemata, Myxophaga, Adephaga. Brill, Leiden, 280–283.

[B22] Häckel M, Farkač J, Wrase DW (2010) A check-list of the tribe Broscini Hope, 1838 of the World (Coleoptera: Carabidae). Studies and Reports, Taxonomical Series 6(1–2): 55–56.

[B23] Jiang R-X, Chen X-S (2023) New species and distributional records of the genus *Broscosoma* Rosenhauer, 1846 (Coleoptera, Carabidae, Broscinae) from Fujian, China. Zootaxa 5244(1): 089–095. 10.11646/zootaxa.5244.1.8 37044476

[B24] Jiang R-X, Feng Q, Wang S (2020) Two new species of the genus *Broscosoma* Rosenhauer, 1846 (Coleoptera, Carabidae, Broscinae) from China. Zootaxa 4821(1): 173–180. 10.11646/zootaxa.4821.1.10 33056338

[B25] Jiang R-X, Liu X-F, Wang S (2021) New species and new distributional record of the genus *Broscosoma* Rosenhauer, 1846 (Coleoptera, Carabidae, Broscinae) from China. Zootaxa 4951(1): 137–146. 10.11646/zootaxa.4951.1.6 33903417

[B26] Kavanaugh DH, Liang H-B (2021) Inventory of the Carabid Beetle Fauna of the Gaoligong Mountains, Western Yunnan Province, China: Species of the Tribe Broscini (Coleoptera: Carabidae). Proceeding of the California Academy of Sciences, Series 4 67(4): 85–182.

[B27] Li Z-C, Chen J-H (2022) Supplemental notes on *Broscosoma valainisi* Barševskis, 2010 (Coleoptera: Carabidae: Broscini). Faunitaxys 10(53): 1–5.

[B28] Putzeys JAAH (1877) Carabiques nouveaux du nord de l’lnde (Darjeling). Entomologische Zeitung Stettin 38: 100–103.

[B29] Sciaky R, Facchini S (2005) Revision of the Chinese *Broscosoma* Rosenhauer, 1846, with descriptions of two new species (Coleoptera: Carabidae, Broscinae). Koleopterologische Rundschau 75: 1–12.

[B30] Semenov AP (1900) Rod *Broscosoma* Putz. (Coleoptera, Carabidae), ego vidy i ikh geograficheskoe raspredelenie. Horae Societatis Entomologicae Rossicae 34: 74–87.

